# Effect of tungsten doping on the structural, morphological and bactericidal properties of nanostructured CuO

**DOI:** 10.1371/journal.pone.0239868

**Published:** 2020-09-28

**Authors:** Angela Mercedes Raba-Páez, João Otávio D. Malafatti, Carlos Arturo Parra-Vargas, Elaine Cristina Paris, Miryam Rincón-Joya

**Affiliations:** 1 Department of Physics, Universidad Francisco de Paula Santander, Cúcuta, Norte de Santander, Colombia; 2 Department of Physics, Universidad Pedagógica y Tecnológica de Colombia, Tunja, Boyacá, Colombia; 3 Department of Chemistry, Universidade Federal de São Carlos, São Carlos, São Paulo, Brasil; 4 EMBRAPA Instrumentação, São Carlos, São Paulo, Brasil; 5 Department of Physics, Universidad Nacional de Colombia, Bogotá, Cundinamarca, Colombia; University of California Santa Barbara, California, USA, INDIA

## Abstract

Copper oxide (CuO) has been broadly used in different technological and biological applications. However, based on the literature review, there are few reports describing the synthesis of tungsten doped copper oxide and its biological applications, although CuO and W (tungsten) based nanomaterials have been reportedly already synthesized. In this study we synthesized novel CuO and CuO/W (at.1%, 2% and 4%) nanoparticles and explored their tungsten content-dependent bactericide activity. In order to obtain the materials, was used a co-precipitation method which is of low cost. The synthesized materials were characterized by x-ray diffraction (XRD); XRD results indicated that only the sample with at.1% of W presented pure Tenorite phase. Diffuse reflectance spectroscopy (DRS) allowed to obtain the band gap energy values; CuO/W (at.2%) sample exhibited the minimum value of 2.62 eV. Grains sizes ranging from 39.78 to 53.47 nm were established through field emission—scanning electronic microscopy (FE-SEM), and these sizes were confirmed by transmission electron microscopy (TEM). Doping with W also influenced the morphology obtained in all cases. BET (Brunauer, Emmett, Teller) analysis allowed to establish an increase in specific surface area and pore size with W doping. The particle size was determined by dynamic light scattering (DLS). The bactericidal properties were tested using well diffusion method for *Escherichia coli* and *Staphylococcus aureus* bacteria. Bactericide response of CuO nanoparticles was improved by the inclusion of W dopant into the CuO structure, leading to an expansion in the inhibition zone for the CuO/W (at.1%) sample; inhibition halo diameters were 1.5 and 12 mm for CuO and CuO/W (at.1%), respectively. Hence, it was possible to infer the remarkable importance of the crystalline phase, morphology, particle size and specific superficial area of the CuO/W (at.1%) nanoparticles in its bactericide performance. WO_3_ secondary phase affected the bactericide response of the materials obtained at at.2% and at.4% of tungsten content.

## Introduction

Nanoparticles and nanocomposites of metallic oxides have showed excellent antimicrobial activity. Among these, ZnO nanoparticles and modified ZnO nanoparticles by adding noble metals such as Ag. The deposition of Ag nanoparticles on a semiconductor metallic oxide surface can increase the interfacial charge-transfer kinetics to metals and semiconductors, which can lead to an increase in the Ag doped ZnO nanoparticles antibacterial properties. Panchal et al. [1] disclosed the green synthesis process of ZnO nanoparticles and Ag/ZnO nanocomposites by means of *Ocimum tenuiflorum* plant extract. They obtained 1.0% Ag/ZnO nanocomposites with hexagonal structure, size ranging between 14 and 22 nm and excellent antimicrobial performance. In another study, the photocatalytic and bactericide activities against *Escherichia coli* and *Klebsiella* bacterias of magnesium oxide (MgO) and zinc oxide (ZnO) nanoparticles, and ZnO/MgO nanocomposites were evaluated [2]. There, ZnO/MgO nanocomposites exhibited an enhanced antibacterial activity, compared to the pure oxides, which proved the advantage of this system.

Copper oxide is a p-type semiconductor of narrow indirect band gap energy that can differ in bulk between 1.2 and 1.5 eV [3]. CuO has physical and chemical properties at nano-level that vary from its micro-level properties. This explains why the number of works related to synthesis of nanostructured CuO has grown as to improve its properties by controlling its morphology and size. Additionally, CuO has been thoroughly studied given its promising applications in enzyme-free glucose electrochemical sensor [4–6], gas sensor [7,8], photocatalysis [9–12], superhydrophobic surfaces [13], removal of organic pollutants in wastewater, arsenic [14] and in the evaluation of its toxicity [15].

Toxicological researches have demonstrated an increase in nanoparticles toxicity as opposed to micrometer size particles of the similar design. CuO nanoparticles are strongly toxic to people. They show a high cytotoxic potential and are hugely toxic in contrast to CuO micrometer-sized and other metallic oxide nanoparticles. Karlsson et al. [16] compared the toxicity of nanoparticles- and micrometer size particles of iron oxide (Fe_2_O_3_, Fe_3_O_4_), titanium dioxide (TiO_2_) and CuO. TiO_2_ micrometer particles caused larger DNA damage as compared to nanoparticles. Fe_2_O_3_ and Fe_3_O_4_ evidenced low toxicity. It was not possible to determine any difference regarding particle size. Hence, the elevated CuO nanoparticles toxicity revealed that this nanolevel dimension may cause a good performance [16]. In another study, Karlsson et al. [15] compared CuO, TiO_2_, ZnO, CuZnFe_2_O_4_, Fe_3_O_4_ and Fe_2_O_3_ nanoparticles cytotoxicity, and the potential to produce DNA harm and oxidative stress with multi-walled carbon nanotubes [15]. The results of this study proved that CuO nanoparticles were more robust with regard to cytotoxicity and DNA damage. The carbon nanotubes exhibited cytotoxic activity causing DNA harm in the slightest dose proved. Additionally, Rousk et al. [17] demonstrated nano scale-CuO toxicity great working on soil bacteria and nontoxicity of the micro- scale CuO. This result also suggests that nanoparticles with size from 1 to 100 nm can be much more noxious than larger particles, when is high the metal dissolution. On the other hand, CuO nanoparticles ranging between 80 nd 160 nm were obtained by damp chemical method and their bactericide activity against *Klebsiella pneumoniae*, *Pseudomonas aeruginosa*, *Salmonella paratyphi* and *Shigella* bacteria was tested [18]. These nanoparticles were smaller than the bacteria pore size and therefore, they can penetrate the cell membrane. According these results, CuO nanoparticles can create stable compounds with essential enzymes interior cells that impede cellular operating, and leading to cell death [18].

CuO has been doped with Pt and Ag to enhance their sensivity properties [19] and with Mn in magnetic studies and sensitivity applications [20]. It has also been doped with Sn to study the Kirkendall effect [21]. With regard to CuO nanostructures doped with transition metal for biological applications, Wang et al. [22] obtained Pd (IV)-doped CuO nanofibers (PCNFs) and undoped CuO nanofibers through electrospinning, and studied the amperometric direct reaction to glucose. PCNFs altered electrodes exhibited exceptional selectivity, reproducibility and stability. Regarding toxicity evaluation, Malka et al. [23] synthesized zinc-doped copper oxide nanostructures and at the same time deposited them on cotton made by means of ultrasound irradiation. Experimental conditions optimization, the reagent proportion, and the precursor concentration led to the obtaining of regular nanoparticles with size about 30 nm. The bactericide activity of the Zn-doped CuO in a colloidal solution or deposited on cotton was evaluated against *Escherichia coli* and *Staphylococcus aureus* bacteria. After 10-minute exposure, a considerable enhancement of 10000 times in the antibacterial activity of the Zn–CuO nanocomposite in contrast to pure CuO and ZnO nanoparticles was noticed. Comparable responses were observed against multidrug-resistant bacteria, in addition underlining the potency of this system. The structural and antibacterial properties of zirconium doped copper oxide nanoparticles were reported in [24]. The antibacterial activity with *Enterococcus faecalis*, *Streptococcus mutans*, *Escherichia coli* and *Stenotrophomonas maltophilia* was evaluated, and the structural analysis indicated that the Zr dopant was included into the CuO structure. This work demonstrated that by rising Zr content in the CuO lattice, bactericide response is enhanced. ROS (free radicals of hydroxyl, OH^•^, hydrogen peroxide, H_2_O_2_, anion superoxide radicals, O_2_^•-^, and singlet oxygen, ^1^O_2_ [24]) creation was established as the main mechanism of the antibacterial performance of the obtained materials. Thakur and Kumar [25] studied the influence of Ag, Co co-doping on the structural properties and antibacterial response of CuO, where XRD analysis verified the presence of unmixed monoclinic crystalline structure of obtained nanoparticles. Additionally, the morphology was established to modify from nanorods to grain-like nanostructures, and afterwards to nanospheres exhibiting alteration of Co content. The nanoparticles antibacterial efficiency was tested against *Staphylococcus aureus*, *Bacillus subtilis*, *Escherichia coli*, and *Pseudomonas aeruginosa*. The study conclusions demonstrated that the co-doped nanostructures were more efficient against the examined microorganism. Kumar et al. [26] synthesized CuO and Zn-doped CuO nanostructures by means of a not complex low-cost sol-gel procedure. The FE-SEM results verified the creation of bunched cauliflower structures after zinc doping. Zn-doped CuO materials showed excellent bactericide performance against *Escherichia coli* and *Staphylococcus aureus* exhibiting a modification in antibacterial response compared to undoped CuO. In summary, reports that describe the synthesis, characterization, and biological applications of nanostructured copper oxide doped with tungsten are scarce at present.

In this research, we report a novel and simple low-cost co-precipitation technique to produce copper oxide and copper oxide doped with tungsten nanostructures. The co-precipitation method was selected as it promotes the precipitation of interest system (CuO) in the presence of another cation (W). The tungsten content in the synthesized materials was controlled as to determine its influence on the CuO structural, optical, morphological, superficial and bactericidal properties. In general, the bactericidal properties answer to the crystalline phase, the agglomerate size and the specific surface area of the obtained materials. Bactericide activity in CuO/W nanostructures was tested against *Escherichia coli* (*E*. *coli*) and *Staphylococcus aureus* (*S*. *aureus*). Evidences confirm that unique phase, smaller particle size and larger surface area of CuO/W (at.1%) nanoparticles play a relevant role in its bactericide performance.

## Experimental

### Preparation of samples

CuO and CuO/W nanostructured materials were obtained by co-precipitation technique. In the precursor precipitation reaction, copper (II) nitrate 3-hydrate (Cu(NO_3_)_2_∙3H_2_O Synth 96%) was used for the CuO synthesis. Additionally, sodium tungstate dihydrate (Na2WO4∙2H2O Neon 99%) was used to obtain CuO/W at.1%, 2% and 4% precursor. Sodium hydroxide (NaOH Synth 97%) solubilized completely in distilled water was used as precipitating agent. The resulting precipitates were rinsed with distilled water, and later washed with absolute ethyl alcohol (Reatec C_2_H_6_O 99%), and dried in a stove at 60°C for 24 h. Thermal treatment of 500°C was used for the final crystallization.

### Characterization of samples

Crystalline structure of the materials was analyzed through a Shimadzu XRD6000 X-ray diffractometer (Cu K_α_ radiation, λ = 1.5488 Å); 30 mA, 30 kV, 2θ between 10° and 80°, step scan of 0.02° and 1°/min were the operating conditions. The reflectance and absorption coefficient measurements were obtained from diffuse reflectance spectroscopy with a Shimadzu UV-2600 UV-vis spectrophotometer. Field emission—scanning electronic microscopy was utilized to investigate the morphology and to estimate the size of obtained agglomerates. The images were obtained with a ZEISS equipment operating at 5.0 kV and 200.0 nA; the samples were not coated. Elemental mapping by energy dispersive spectroscopy (EDS) was carried out since FE-SEM technique. Transmission electron microscopy images were acquired through a Tecnai F20 Super-Twin TMP FEI equipment operated at an accelerating voltage of 200 kV; the samples for TEM analysis were dissolved in absolute ethanol and afterwards deposited on the copper grids. Specific surface area (S_BET_) of the materials was established by means of BET analysis through a Micromeritics ASAP 2020 particle size analyzer. Dynamic light scattering analyzes was used to estimate the size in suspension of the obtained materials. Zeta potential analyzer Zetasizer Nano S Malver Instruments equipment was used at room temperature by employing a wavelength of 633nm and an incidence angle of 173°.

### Bactericidal assays

Bactericidal activities of the synthesized CuO and CuO/W nanoparticles were tested using Gram-negative bacteria (*E*. *coli*) and Gram-positive bacteria (*S*. *aureus*) following the disc diffusion method. The bacteria were cultured in a mixture of Mueller Hinton Broth culture medium and AGAR as solidifying agent. Bacterial lawn was obtained by spreading 30 mL culture broth as solid nutrient agar plates. After solidification, 100 μL of bacteria solution was poured into each plate. After that, three samples of nanoparticles were immediately placed in the plates. Afterwards, the plates were taken brought to the stove at 37°C for 24 hours. After incubation, the inhibition zone size was measured.

## Results and discussion

### Structural and optical analysis

**Fig 1:**
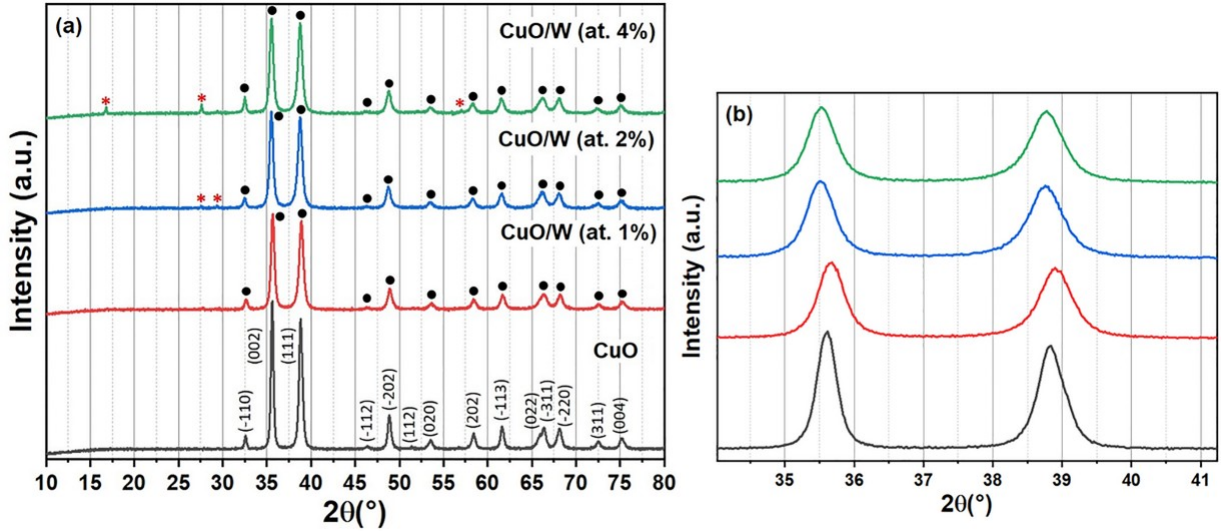
XRD pattern. (a) XRD pattern of copper oxide and copper oxide doped with tungsten. CuO and WO_3_. (b) Magnified XRD spectra.

XRD patterns of the bulk CuO and CuO/W are presented in Fig 1(A). CuO XRD pattern exhibits characteristic peaks with 2θ values at 32.61°, 35.64°, 38.82°, 46.36°, 48.85°, 51.45° 53.58°, 58.37°, 61.59°, 65.92°, 66.36°, 68.20°, 72.49° and 75.08°; these values are assigned to the reflection lines of CuO monoclinic structure -Tenorite phase- PDF No. 00-045-0937 (Powder Diffraction Standards), with a space group C2/c. CuO/W (at.1%, 2% and 4%) diffractograms also exhibits peaks due to this structure. Thermal treatment at 500°C was suitable to obtaining the CuO monoclinic structure. CuO/W (at.1%) XRD pattern does not present any impurity peak, showing the single phase formation. CuO/W (at.2%) diffractogram presents two additional peaks (at 27.55° and 29.40°) and CuO/W (at.4%) diffractogram has, in turn, three peaks (at 16.84°, 27.61° and 57.04°) due to WO_3_ monoclinic structure (PDF No. 01-087-2395). CuO crystalline structure is preserved at at.1% of tungsten content. WO_3_ secondary phase appears at at.2% and at.4% of tungsten content, which may indicate that some tungsten was incorporated into CuO and some more was segregated at secondary phase at these doping values. Diffraction peaks width for the CuO/W (at.2%) sample slightly increased as compared to those of CuO, indicating the formation of smaller particles, which was later corroborated through FE-SEM and TEM analyses.

Fig 1(B) presents the magnified XRD spectra. As a result of the likely W doping residual stress, (002) and (111) peaks slightly move to lower 2θ values, that may lead to anisotropic reduction of crystal lattice and therefore cause lattice deformation, as reported by [27]. Tensile stress leads to the shift to lower angles. Theoretically, this alteration to lower 2θ is caused by the tensile stress created in the CuO structure after doping [26], in this case, presenting various W content.

The crystallite size (*L*) was estimated by means of the Debye Scherrer’s equation, Eq (1). These values are recorded in Table 1. *0*.*94* is the form factor or Scherrer constant, λ is the wavelength, *B* corresponds to the Full Width at Half Maximum, FWHM, and θ is the Bragg diffraction angle. The chosen line to estimate *L* corresponds to the maximum intensity in the diffractogram.

$$L=\frac{0.94\lambda }{B\cos\theta }$$ (1)

**Table 1:** Structural parameters.

	L(nm)	FWHM			d (Å)			Lattice parameters (Å)			V(Å^3^)	ε (x 10^−2^)
Sample		(-110)	(002)	(111)	(-110)	(002)	(111)	a	b	c		
**CuO**	32.60	0.138	0.315	0.256	2.758	2.531	2.330	4.744	3.414	5.133	81.98	0.2248
**CuO/W (at.1%)**	38.90	0.315	0.264	0.168	2.753	2.528	2.327	4.406	3.558	5.127	79.26	0.1812
**CuO/W (at.2%)**	37.21	0.276	0.276	0.236	2.767	2.537	2.339	4.479	3.549	5.145	80.65	0.2138
**CuO/W (at.4%)**	34.73	0.138	0.295	0.098	2.767	2.537	2.333	4.205	3.715	5.145	79.26	0.2277

Crystallite size (*L*), full-width at half maximum (FWHM), inter-planer spacing (*d*), lattice parameters (*a*, *b*, *c*), volume of the unit cell (*V*) and strain (ε) of CuO and CuO/W nanostructures.

CuO/W (at.1%) sample has greater crystallite size than undoped CuO. The tungsten doping ion inclusion at at.1% in the copper oxide crystalline structure led to formation of a greater crystal. Crystallite size values were smallers at at.2% and at.4% of tungsten concentration, as compared to at.1%, which confirmed that some tungsten was incorporated into the CuO crystalline structure and some portion was segregated as well.

The lattice parameters (*a*, *b*, *c*), the volume of the unit cell (*V*) and the strain (ε) of the obtained samples were estimated through the Eqs (2)–(4) [24,28] and the results are presented in Table 1. *d* corresponds to the distance between the crystallography planes and *h*, *k*, *l* are the miller indices.

$$\frac{1}{d^{2}}=\frac{1}{\sin^{2}\beta }\left(\frac{h^{2}}{a^{2}}+\frac{k^{2}}{b^{2}}\sin^{2}\beta +\frac{l^{2}}{c^{2}}-\frac{2hl\;\cos\beta }{ac}\right)$$ (2)

V=abcsinβ (3)

$$\varepsilon =\left(\frac{1}{\sin\theta }\right)\left[(\frac{\lambda }{L})-(B\;\cos\;\theta )\right]$$ (4)

**Fig 2:**
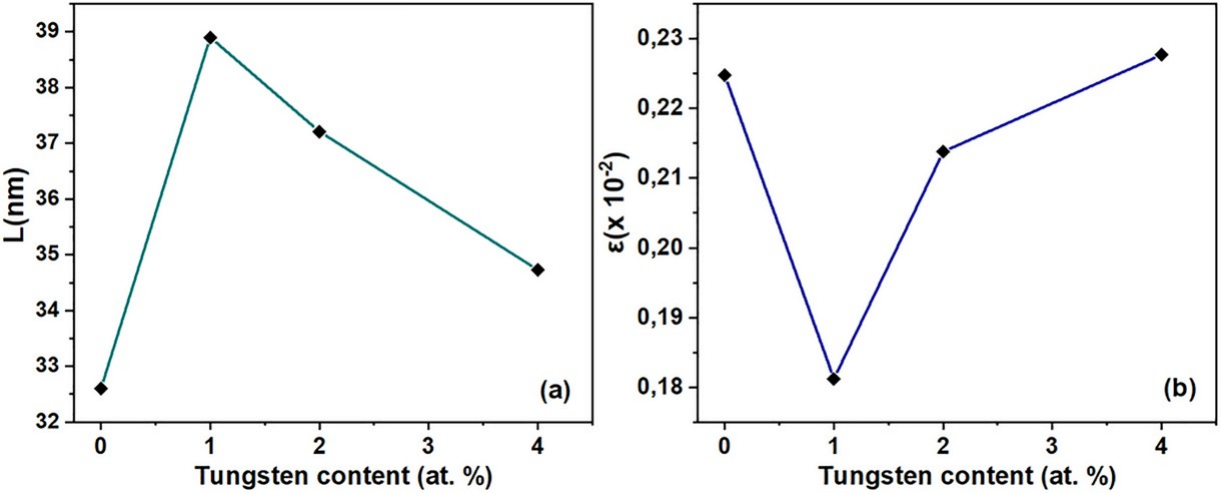
Variation of structural parameters. Evolution of the crystallite size and the strain with the tungsten content.

As seen in Table 1, *a* decreases while *b* increases while enhancing the W content, because of the variation in radius ionic between Cu and W. The tungsten inclusion into the CuO crystal structure is expected to lead to the preferential increase of a lattice parameter and hence, unit cell volume is quasi preserved. The unit cell volume did not exhibit a considerable change, which may be linked to undistorted crystal structures due to the tungsten inclusion. Throughout (-110) direction, FHWM decreases with the increase of tungsten content, which verifies that structural defects decrease with increasing doping due to the tungsten segregation at secondary phase. Variation in crystallite size and strain in the presence of tungsten content is exhibited in Fig 2. Increase in tungsten content resulted in a decrease in *L*, but an increase in the strain. With the inclusion of the tungsten ion, more tension was induced in the CuO crystalline structure.

**Fig 3:**
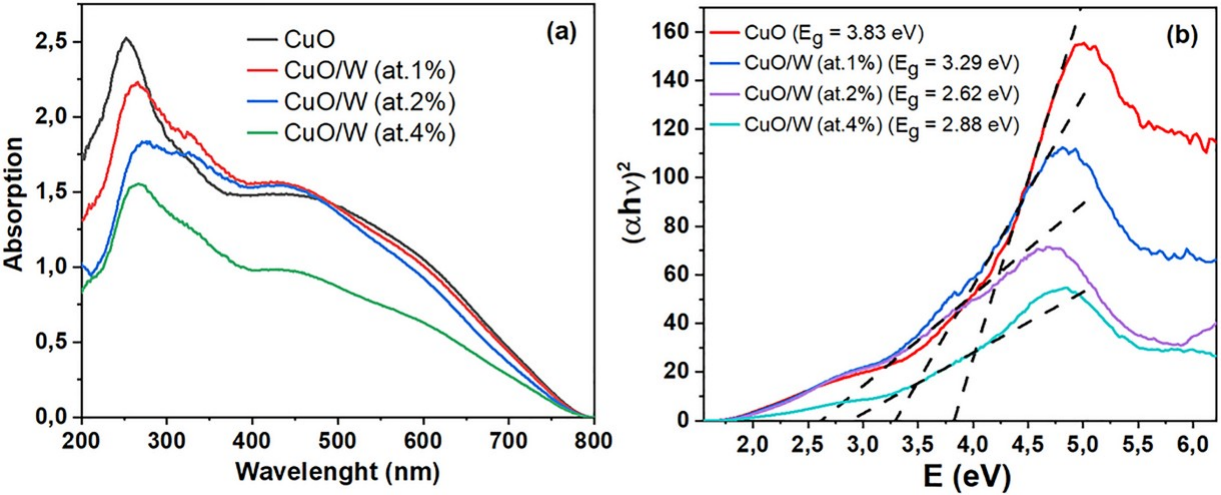
Optical characterization. (a) Variation of absorption coefficient and (b) variation of (αhν)^2^ vs. photon energy of CuO and CuO/W.

Using Kubelka Munk [29] transformation, developed by the equipment software, for reflectance measurements contributed to obtain absorption coefficient (*α*). Absorption behavior of CuO and CuO/W materials is presented in Fig 3(A). Absorption has a tendency to decrease while the wavelength increases, which is current in semiconductors and can occur in lattice deformation due to strain by the inclusion of W ions into the CuO crystal lattice, according to XRD results. The materials evidenced to have absorption peaks centered at 252 nm, 266 nm, 276 nm and 267 nm for the undoped CuO and W doped at at.1%, 2% and 4%, respectively. Hence, a red shift was observed, which may be attributable to impurities, imperfection states, and p-d electrons mixture between oxygen and metal ions [26] generated by tungsten doping. Band gap energy, *E*_*g*_, and the semiconductor absorption coefficient are associated through the Eq (5). There, *hν* is the incident photon energy and *C* is a material dependent constant [30–32]. The *n* value depends on the nature of transition. Direct allowed *n* = 1/2 and indirect allowed *n = 2* [29,33].

$$\alpha =\frac{C{\left(h\nu -E_{g}\right)}^{n}}{h\nu }$$ (5)

The band gap energy was acquired by plotting *(αhν)*^*2*^ vs. *hν* (Fig 3(B)). The intersection between the linear fit and the *hν* axis results in *E*_*g*_. Band gap energy values for the doped samples are lower than that evidenced in CuO. For undoped CuO the band gap is 3.83 eV, whereas *E*_*g*_ for CuO/W decreases as W content increases: 3.29 eV for CuO/W (at.1%), 2.62 eV for CuO/W (at.2%) and 2.88 eV for CuO/W (at.4%). Jiang et al. [27] also reported a reduction in the band gap energy of CuO when increasing the amount of Zn dopant. There, the band gap energy decreased from 3.41 eV (for CuO) to 1.42 eV (for CuO doped with Zn). The authors attributed this red-shift to impurity levels originated by zinc inclusion; a number of defects can bring about intragap defect states and the p–d mixing of oxygen and metal ions [27]. W dopant may produce an impurity band in the CuO band gap, which can combine with the conduction band after tungsten inclusion. This effect may decrease the band gap energy, as it similarly occurs with Zn doped CuO [26] and Ce doped ZnO [34]. For those doped samples, the band gap energy reduces when decreasing the crystallite size, which may be derived from less realignment in orientation and poor crystallinity of the obtained materials, as reported by Purohit et al. [33]. CuO is a p-type semiconductor therefore, the band gap energy decreases due to the carriers reduced number after doping.

### Morphological and surface properties

**Fig 4:**
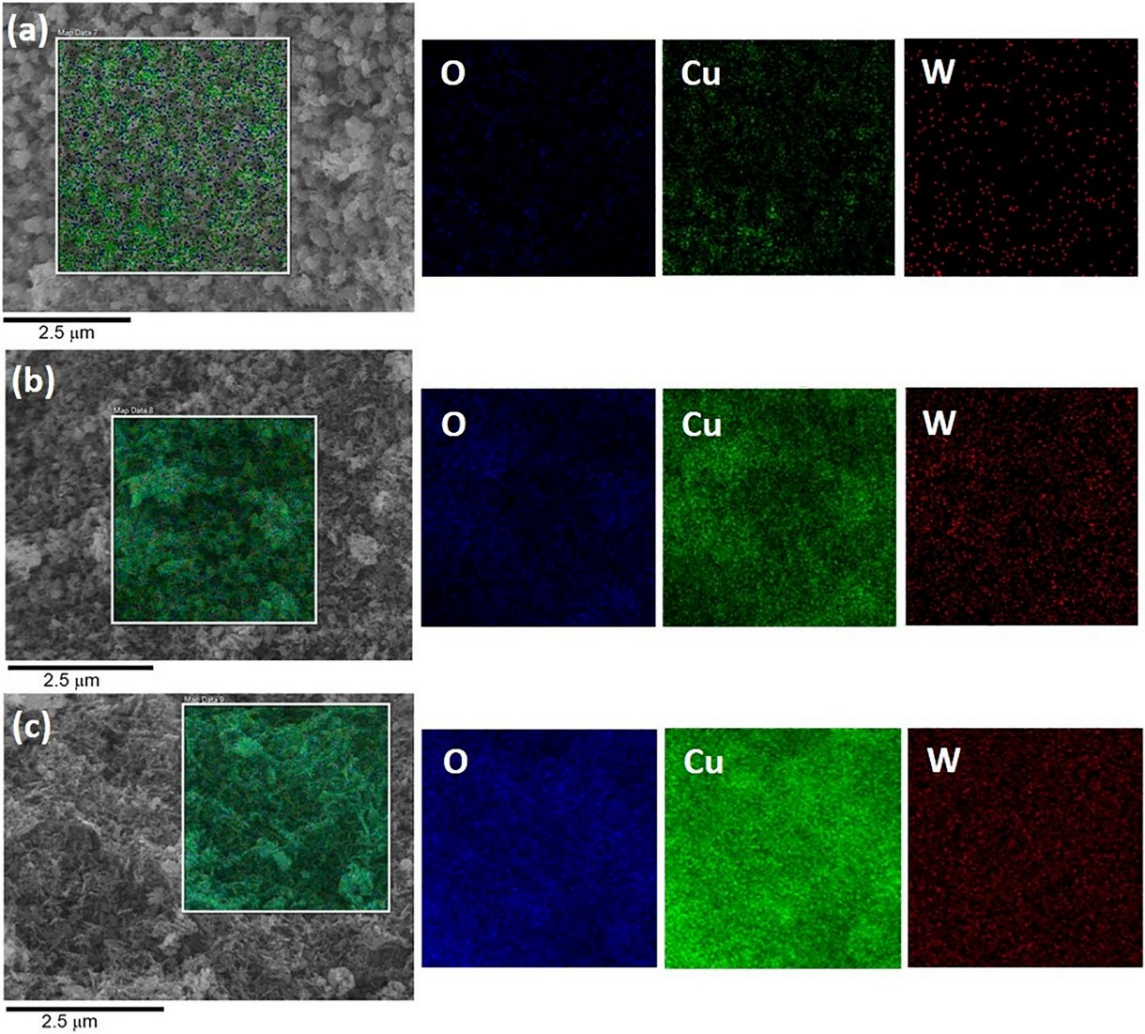
FE-SEM and EDS images. FE-SEM image and elemental mapping of (a) CuO/W (at.1%), (b) CuO/W (at.2%) and (c) CuO/W (at.4%).

The EDS technique was employed to verify the presence of tungsten in the obtained materials. EDS images are shown in Fig 4. EDS images of CuO/W (at.1%, 2% and 4%) samples revealed the presence of O, Cu and W elements in all obtained doped materials.

**Fig 5:**
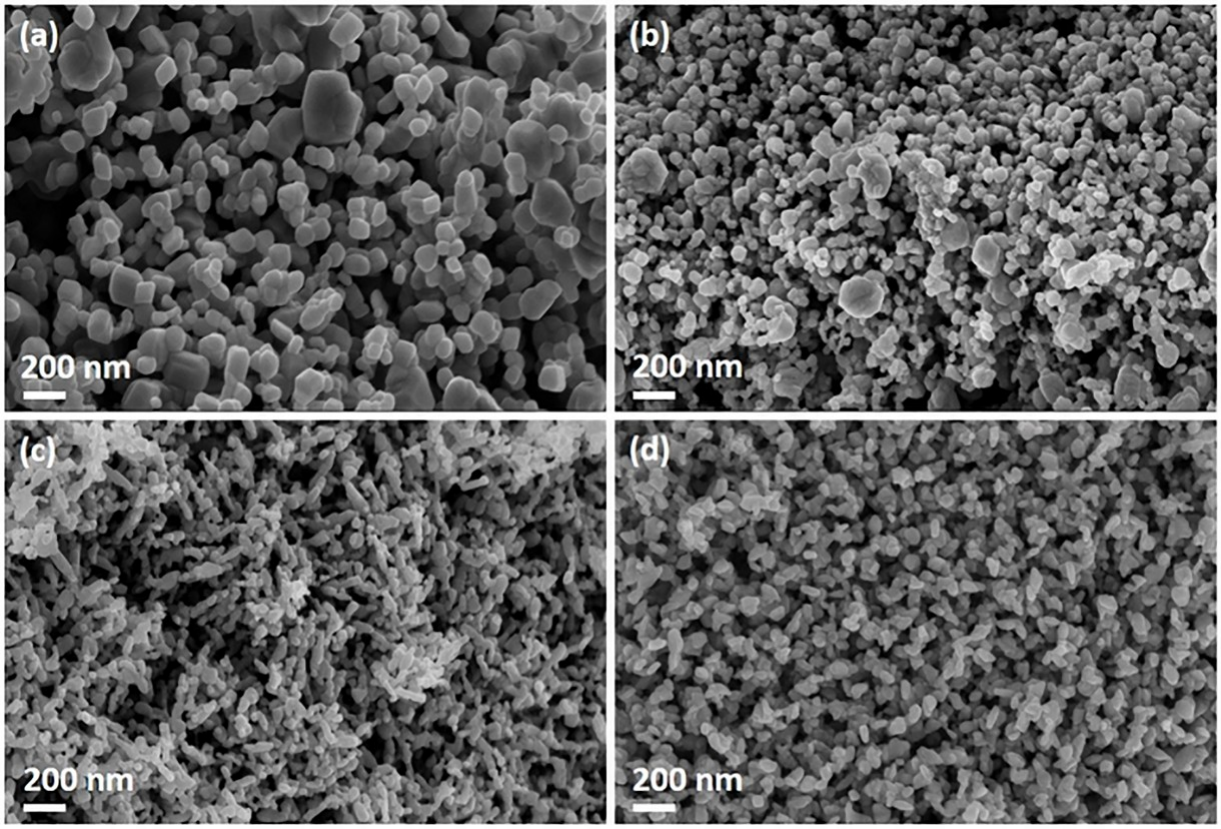
FE-SEM images. FE-SEM images of (a) CuO, (b) CuO/W (at.1%), (c) CuO/W (at.2%) and (d) CuO/W (at.4%). Mag = 100.00 KX.

FE-SEM analysis was carried out to study the morphology and to estimate the grain size of CuO and CuO/W at different tungsten concentrations. FE-SEM image of CuO presented in Fig 5(A) exhibits particles agglomerates formation with regular and well-defined morphology. CuO/W (at.1%) image (Fig 3(B)) shows smaller particles agglomerates formation than the obtained for CuO. Morphological changes of CuO/W (at.1%) materials are linked to the doping ion inclusion. CuO/W (at.2%) image (Fig 5(C)) exhibits rice grain-like structures while CuO/W (at.4%) image (Fig 5(D)) exhibits laminar structures. Morphological changes in these two samples demonstrate that some tungsten was incorporated into CuO and some more was segregated to secondary phase, in accordance with the XRD results. WO_3_ secondary phase inhibited the grain growth at at.2% and at.4% tungsten content. The contact area between the particles is lost by agglomerating the particles. Therefore, an increase in doping concentration influences grain shape in the formation of different morphologies.

**Fig 6:**
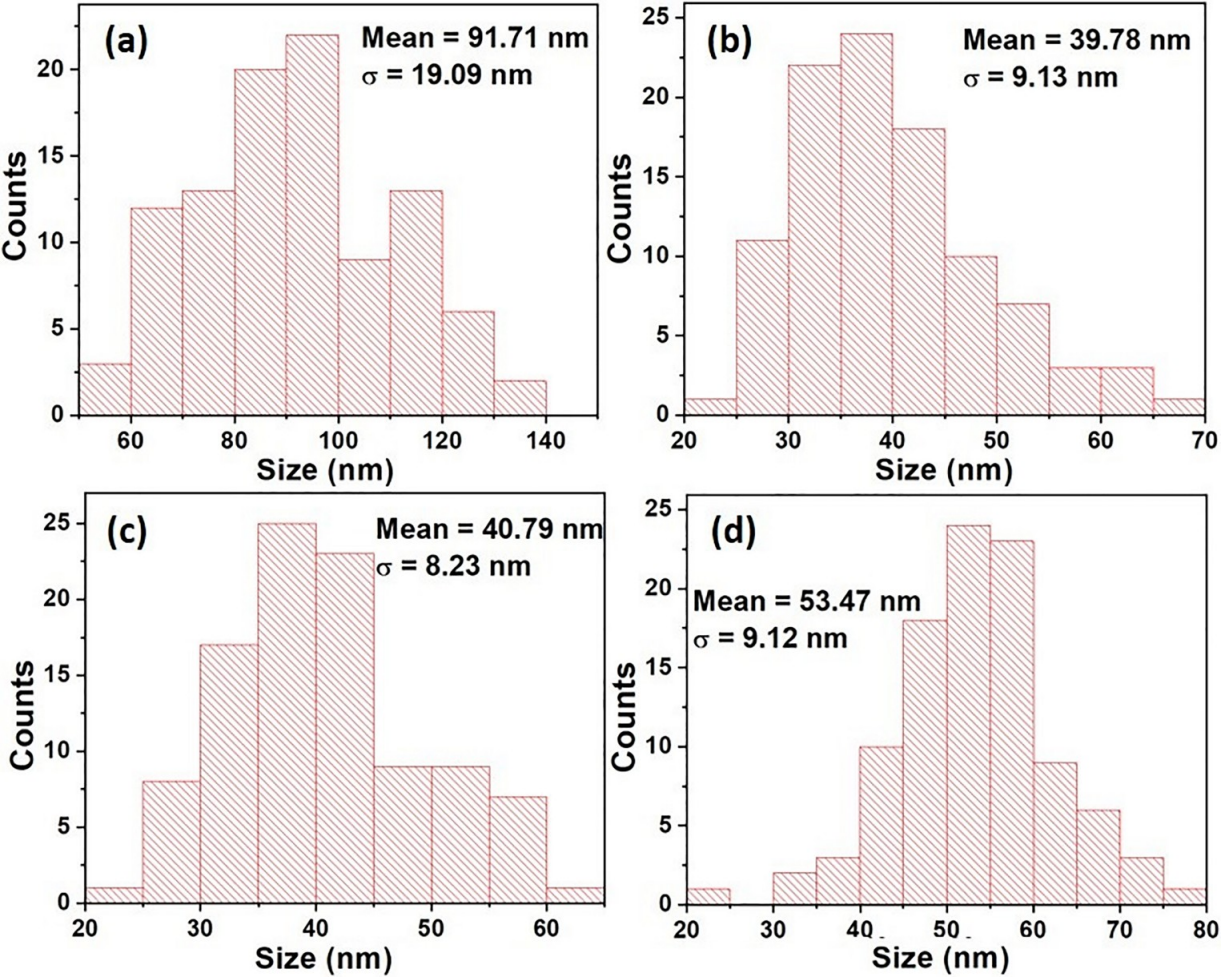
Histograms of the size distribution. Histograms of the size distribution of the (a) CuO, (b) CuO/W (at.1%), (c) CuO/W (at.2%) and (d) CuO/W (at.4%) agglomerates.

Fig 6 presents the CuO and CuO/W (at.1%, 2% and 4%) size distribution histograms. The sizes of the grains were estimated using Image-J software. The estimated size was the grain diameter for the undoped copper oxide and doped at 1% and 4%. Grain length was estimated for CuO/W (at.2%). Tungsten incorporation in the CuO crystalline structure leads to CuO/W (at.1%) grain size decrease. For the CuO/W (at.2%) and CuO/W (at.4%) samples, the estimated dimension was also smaller than for CuO, which confirms that some of tungsten was incorporated into the CuO crystalline structure, according to XRD results.

Bacterial cells have micrometric size; mostly of these microorganisms have cellular membranes composed of pores in the dimension of nanometer range [35]. The synthesized CuO/W nanostructures from this study could cross the cell membranes through such bacterial pores. Thereby, the preparation of CuO/W stable nanoparticles complying with specific nanometric size as to restrict bacterial growth while crossing the cell membrane was of utmost importance. On the other hand, the CuO nanoparticles morphology has, as well, an important role determining its toxicity [36]. Therefore, CuO/W (at.1%) nanoparticles, CuO/W (at.2%) rice-like nanostructures and CuO/W (at.4%) laminar nanostructures could present better toxicity than CuO nanoparticles.

**Fig 7:**
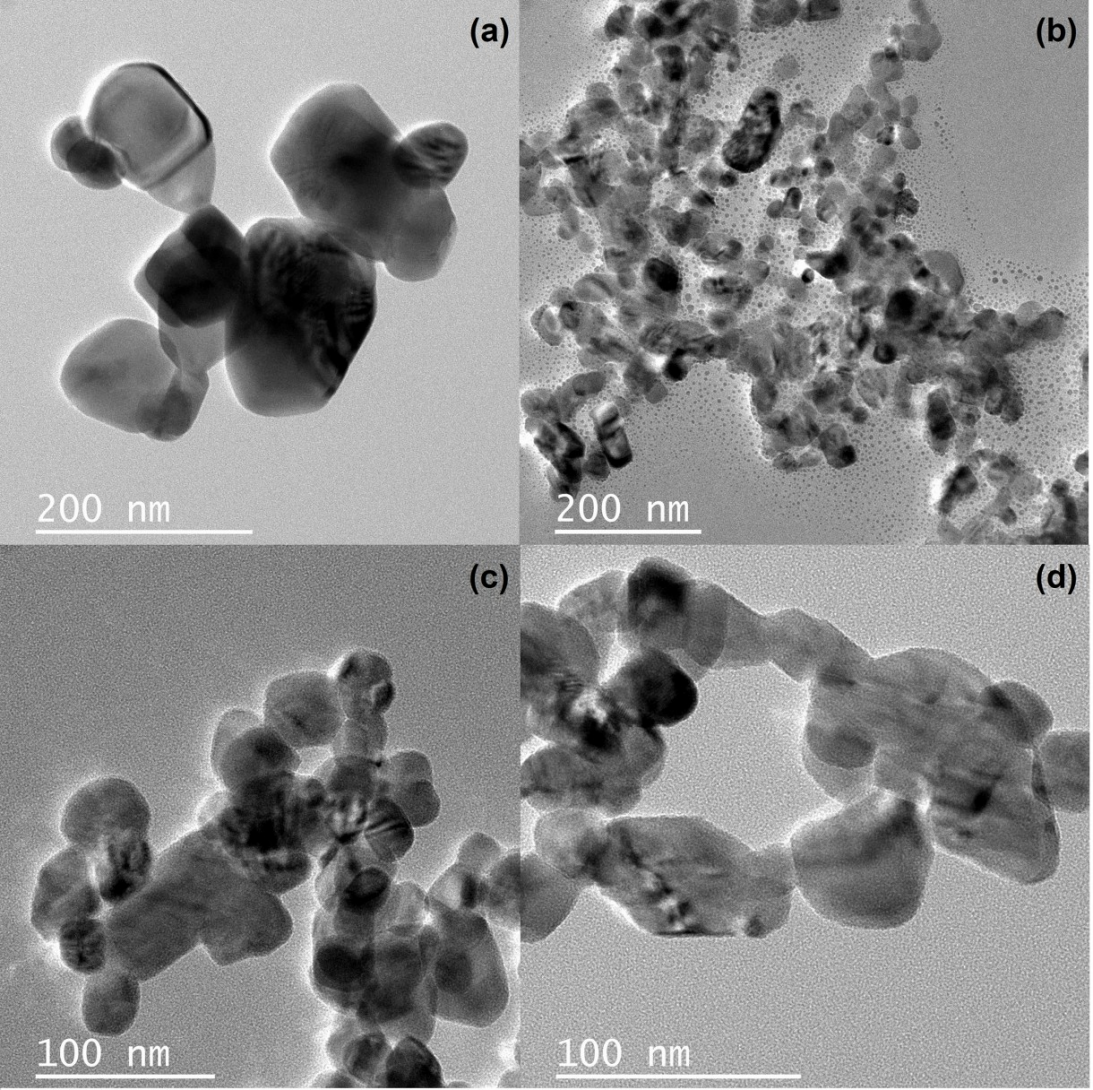
TEM images. (a) Undoped CuO and CuO/W doped at (b) at.1%, (c) at.2% and (d) at.4%.

TEM images of CuO and CuO/W nanostructures are presented in Fig 7. The estimated particle size, according to TEM analysis, are recorded in Table 2. The images show agglomeration of small particles. Fig 7(B)–7(D) exhibit the presence of uniform particles aggregates smaller than CuO particles agglomerates, Fig 7(A). The morphology of undoped CuO nanoparticles changed after tungsten inclusion and the size of the obtained materials decreased as the tungsten content increased, according to FE-SEM results. This result is opposite to the results presented in [25], where the CuO synthesized nanoparticles size increased as the concentration of Ag, Co co-doping increased.

**Table 2:** Parameters of obtained samples.

Sample	CuO	CuO/W (at.1%)	CuO/W (at.2%)	CuO/W (at.4%)
**Grain size**^**a**^ **(nm)**	91.71	39.78	40.79	53.47
**Particle size**^**b**^ **(nm)**	93.62	38.32	37.46	36.69
**S**_**BET**_^**c**^ **(m**^**2**^**/g)**	6.11	12.75	17.69	17.70
**Pore size**^**c**^ **(Å)**	113.81	160.16	175.51	206.60
**Dimension in suspension**^**d**^ **(nm)**	390.23	400.90	264.13	340.03

Grain and particle size, specific surface area, pore size and dimension in suspension.

^a^Using FE-SEM.

^b^Using TEM.

^c^Using BET analysis.

^d^Using dynamic light scattering (DLS).

**Fig 8:**
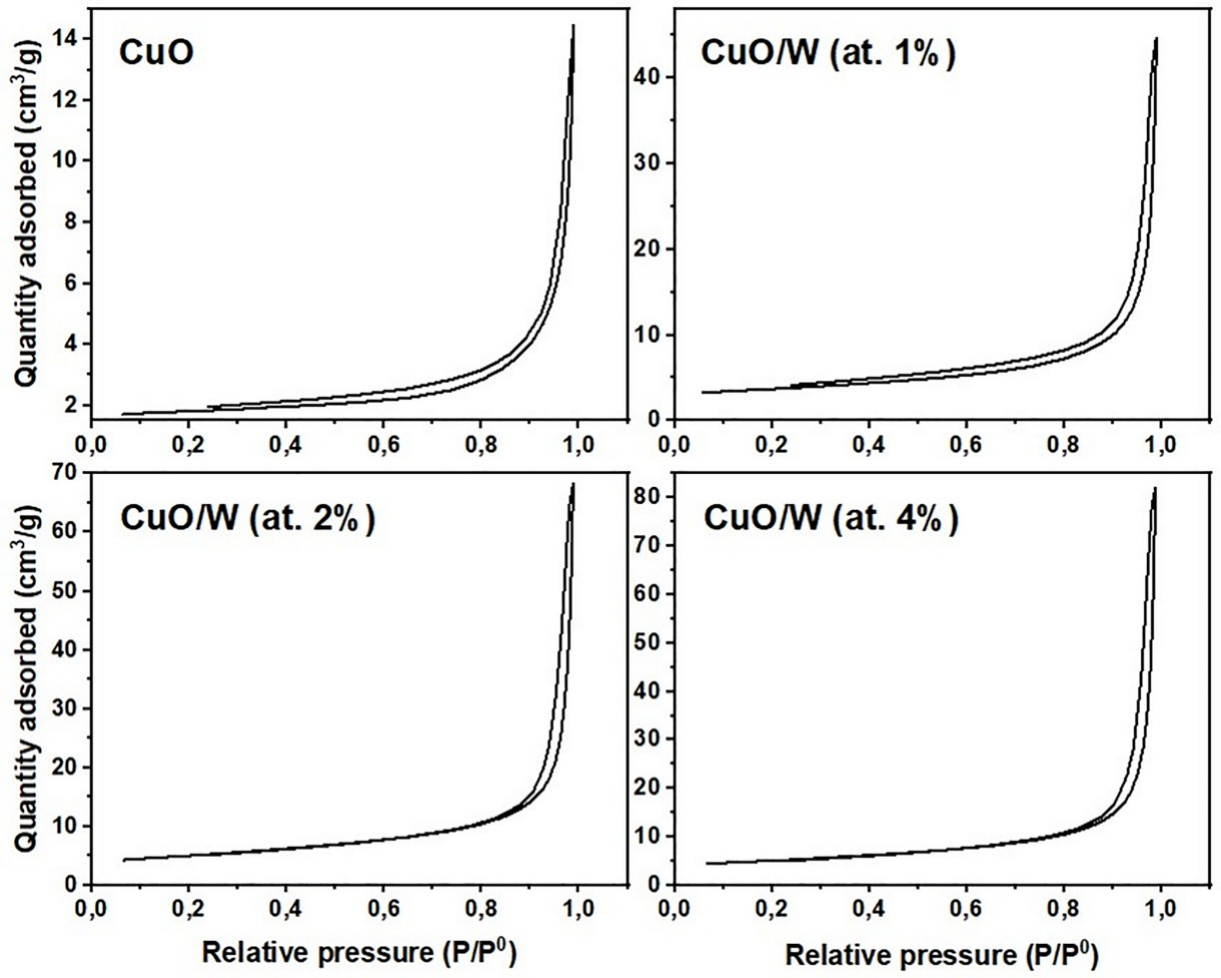
Adsorption isotherms. Adsorption isotherms of the obtained samples.

Fig 8 exhibits the CuO and CuO/W (at.1%, 2% and 4%) materials adsorption isotherms. CuO and CuO/W (at.1%) isotherms have a type-V form; CuO/W (at.2%) and CuO/W (at.4%) isotherms have a type-IV form, according to the classification presented by Alothman [37]. The hysteresis loop form denotes that there are few adsorbate-adsorbent interactions in the bulk materials, which may be caused by solids with large pores. This assumption was later confirmed by the BET method pore size estimation. Results are tabulated in Table 2. Pore size values confirmed that the obtained samples belong to materials of pore size distribution and geometry of a mesoporous sample. Pore size increased from 11 to 21 nm (Table 2) due to tungsten presence. A hysteresis at partial pressures higher than 0.2, due to the mesoporosity, is identified in CuO and CuO/W (at.1%). According to FE-SEM results, this behavior is associated with porous material containing well-defined cylindrical-like pore or agglomerates of uniform roughly spheres, according to reported by Alothman [37]. A hysteresis at partial pressures higher than 0.8 is exhibited by CuO/W (at.2%) and CuO/W (at.4%). These materials have slit-shaped pores and plate-like particle aggregates. Specific surface area, S_BET_, was determined by analyzing data at relative pressures between 0.1 and 1.0. Results are presented in Table 2. S_BET_ results evidenced an increase in tungsten content causes an enhance in the specific surface area. S_BET_ results and FE-SEM and TEM images show that small S_BET_ measurements are associated with larger particle sizes. On the other hand, increased porosity results in enhanced stress which is in accordance with the values obtained for strain, recorded in Table 1.

Surface features may induce more surface-related phenomena, generating more reactive oxygen species and consequently facilitating higher toxicity [36]. The CuO/W (at.1%) surface features from the sample were significantly better as compared to the CuO sample. Hence, the CuO/W (at.1%) sample could have better bactericide performance. Similarly, CuO/W (at.1%) nanoparticles have a large specific surface area, which can increase toxicity considering the presence of reactive atoms and molecules on the surface, in line with Nel et al. [38].

Nanoparticle agglomerates size in isopropanol suspension was estimated using DLS, in view of nanoparticles frequently create agglomerates in a suspension. Results are showed in the Table 2. The observed values were larger than the particle size measured through FE-SEM and TEM for all obtained materials. Hence, nanoparticles formed agglomerates in isopropanol medium. The CuO/W (at.1%) nanoparticles created larger agglomerates, about 10 times the size estimated by FE-SEM analysis.

### Bactericidal tests

**Fig 9:**
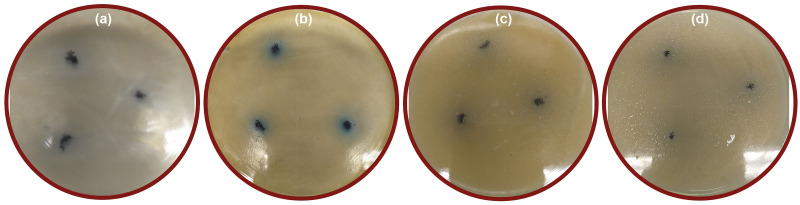
Zone of inhibition results. CuO/W (at.1%) inhibition zone for (a) *E*. *coli* and (b) *S*. *aureus*, and CuO/W (at.4%) inhibition zone for (c) *E*. *coli* and (d) *S*. *aureus*.

**Fig 10:**
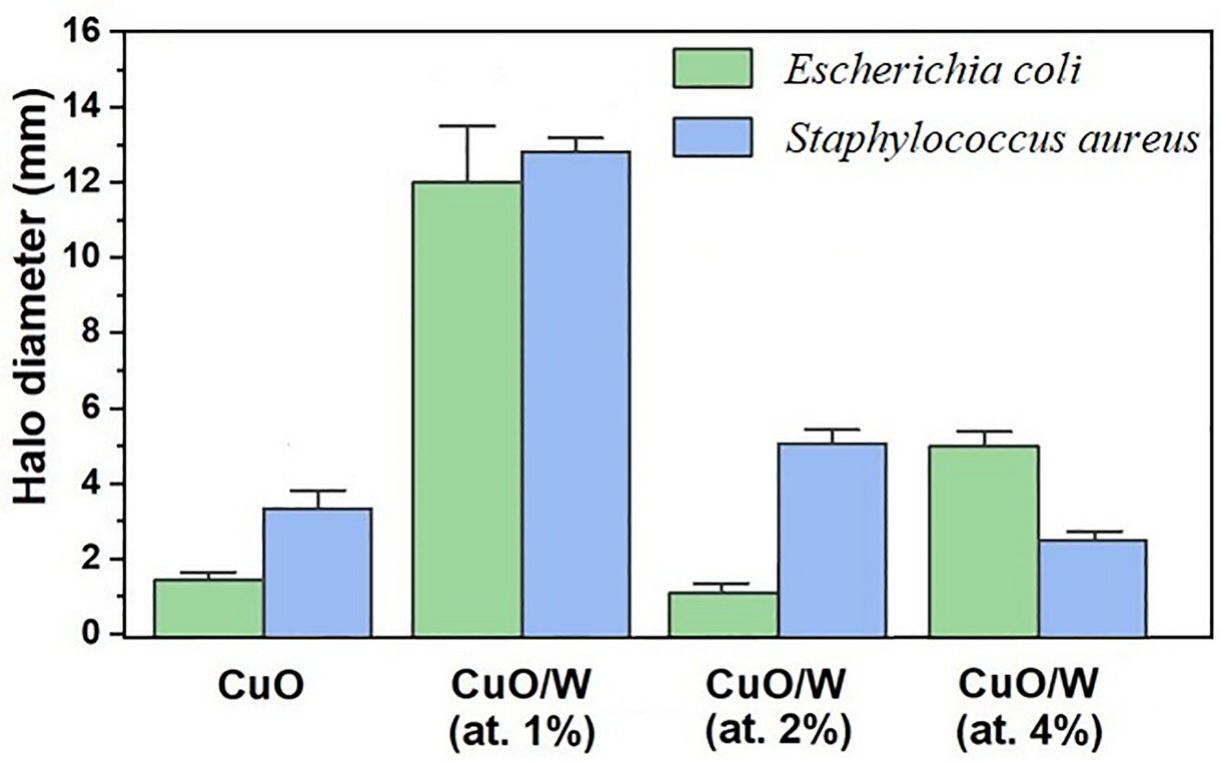
Bar diagram—Inhibition zone. Bar diagram—Inhibition zone in CuO and CuO/W nanoparticles against Gram-negative and -positive bacteria.

Inhibition data are presented in Table 3. The most outstanding inhibition zones are presented in Fig 9. In general, bactericidal properties depend on particle size and specific surface area. These properties have to do with greater retention time for bacterium nanoparticles interaction. Low CuO bactericide response (Fig 10) can be attributed to a low number of soluble metal ions coming from the metallic oxide particles, according to Aruoja et al. [39]. This idea is further developed in Luna-delRisco et al. [40], who attributed that nanoparticles toxicity can only be partially explained by CuO nanoparticles dissolution into Cu ions.

**Table 3:** Bactericide response results.

Sample	Zone of inhibition (mm)	
	*E*. *coli*	*S*. *aureus*
CuO	1.50	3.20
CuO/W (at.1%)	12.00	12.50
CuO/W (at.2%)	1.00	5.00
CuO/W (at.4%)	5.00	2.50

Bactericide response of copper oxide and tungsten-doped cooper oxide.

The CuO/W (at.1%) sample had the largest bactericide response, as stated in the comparison presented in Fig 10. Release of Cu ions was more feasible for this sample due to the less presence of strain This behavior can be attributed to absence of secondary phase, particle size smaller as compared to the CuO, S_BET_ double of the CuO, pore size larger than the CuO and dimension in suspension similar to CuO. These novel properties led to results better in the bactericide response for the obtained materials set. Although the CuO/W (at.2%) and CuO/W (at.4%) samples evidenced very regular morphology (rice-like and plate-like structures, respectively) and larger specific surface areas than CuO/W (at.1%), their bactericide performance was not very high. This behavior may be related to the presence of WO_3_ secondary phase due to tungsten segregation.

CuO/W (at.1%) particle size has a direct function in its bactericide response. The surface-to-volume ratio increments when the particle size is reduced towards the -nano- size then, size effects related to nanoparticles become more noticeable [3]. On the other hand, CuO/W (at.1%) toxicity can also be explained by its high specific surface area, which leads to high surface reactivity. Dey et al. [36] raised two assumptions for toxicity originated by CuO nanoparticles: (1) the production of reactive oxygen species and (2) the induction of oxidative stress. Regarding the first paradigm, this leads to availability of chemically reactive functional groups on the material surface that would have an important function in its bactericide response. Consequently, large specific surface areas of CuO/W (at.1%) nanoparticles cause higher bactericide response than CuO in bulk. The strong antibacterial activity of CuO/W (at.1%) nanoparticles may also be due to reactive oxygen species production by the nanoparticles connected to the bacterial cells, which causes an increase of the intracellular oxidative stress [41]. In such process, oxy radicals could be generated in CuO/W (at.1%) water suspensions and in agree with this hypothesis, toxic oxidative stress may generate an extensive range of mitochondrial perform, including interruption of electron flow in the inner membrane, dissemination of the mitochondrial membrane potential and mitochondrial Ca^2+^ absorption [36]. In this way, the key mechanism of CuO/W (at.1%) bactericide response may be the capacity of doped oxide to damage the mitochondria [16]. Results of Fig 10 also show a slight improvement in the inhibition of *S*. *aureus*; therefore, *E*. *coli* is less responsive to the CuO/W (at.1%) nanoparticles. This could be explained by the *E*. *coli* lipid bilayer cell membrane. Thus, this work suggests that the doping modification in CuO with W in pure phase would be favor the bactericide efficacy of the material.

## Conclusions

CuO and CuO/W nanostructured materials were obtained by co-precipitation method with controlled phases and morphologies through tungsten content control and final thermal process. XRD results confirmed that tungsten cation can be incorporated in the copper oxide crystalline structure, without the presence of secondary phase at at.1% of tungsten content and with the WO_3_ secondary phase at at.2% and at.4% of tungsten content. The study of structural parameters showed a reduction in the crystallite size and an increase in the strain, when the tungsten content was increased. Optical analysis revealed a reduction in the band gap energy value, from 3.83 eV for CuO to 3.29 eV for CuO/W (at.1%) sample. This change in the band gap energy can be related to the variation in crystallinity of CuO with the tungsten inclusion. Additionally, it was verified the variation in the band gap energy with changes in the CuO/W grain sizes. FE-SEM images exhibited changes in the nanoparticles morphology as a consequence of tungsten incorporation in the CuO, and the decrease in grain size from 91.71 nm for CuO to 39.78 nm for CuO/W (at.1%) sample. TEM images allowed to verify that particle size was smaller for doped samples than undoped CuO. S_BET_ (12.75 m^2^/g) and pore size (160.16 Å) of the sample obtained at at.1% of tungsten content increased because of tungsten presence. The bactericide activity of CuO/W nanoparticles was established to be subject to tungsten content. CuO/W (at.1%) nanoparticles exhibited novel properties in phase, morphology, particle size and specific superficial area, which were optimal in its bactericide performance. Novel CuO/W (at.1%) nanoparticles evidenced a better bactericide performance than CuO nanoparticles, showing an inhibition zone of 12 mm. Cu ions releasing was more feasible at 1% of tungsten content due to minor strain.
